# Supplementary material for: Measuring the Autistic Women’s Experience (AWE)
Source: Int J Environ Res Public Health. 2023 Dec 6;20(24):7148. doi: 10.3390/ijerph20247148 (PMC10742621; doi:10.3390/ijerph20247148)
Supplement: Supplementary file 1 [file ijerph-20-07148-s001.zip › Supplementary Materials S1.pdf]

Supplementary Materials S1: Full-length item list

New items developed for AWE

All 52 newly developed items are listed below. A formatted final version of the AWE can be freely downloaded from the Supplementary files (www.mdpi.com/xxx/s1) and in Dutch from emfasisonderzoek.org (please cite this publication when using it).

* = items are reverse scored; ~~Strikethrough~~ = items were removed after discriminant and factorial analysis

1. ~~Other people often don't understand what I am trying to say*~~
2. I go to social events when I don't want to because it's expected of me*
3. ~~I observe how people behave in detail*~~
4. Contact with other people gives me energy
5. ~~Other people sometimes say that I meddle too much*~~
6. I know everything about one subject, for example about animals, diets, historical periods or upbringing*
7. ~~I burst into tears if I have too much on my mind*~~
8. I usually adapt to the other person in a friendship*
9. I have enough energy to do normal daily activities
10. ~~I strongly identify with my favourite character, from a film, series, game or book for example*~~
11. I am in touch with my body and rest when I need to
12. ~~After a busy day I have to do nothing for a few days*~~
13. ~~I am fascinated by fashion, appearances, and/or make-up*~~
14. ~~I often forget to eat or drink*~~
15. ~~Other people say that I come across as social in initial contact with them, but that really doesn't happen automatically*~~
16. ~~I find contact with other people very tiring *~~
17. I am good at keeping up friendships
18. ~~I don't pay attention to how I come across in a conversation~~
19. I feel that I really fit in during a conversation
20. After a few days of doing nothing, I really have to do something
21. ~~I find it easy to express my feelings~~
22. ~~I'm an easy eater and like almost everything~~
23. ~~I have periods of physical pain and/or symptoms without a clear cause*~~
24. ~~I can't stand it when the atmosphere in the group is not good*~~
25. ~~I continually check whether my clothes or hair still look okay*~~
26. I notice when I am exhausted or overloaded in time
27. I don't have to think about how I'm going to act toward others
28. I go to social events when I don't want to because I'm afraid I won't belong otherwise*
29. I get upset when someone else's mood isn't good*
30. ~~I stop eating or drinking when I'm full~~

31. After a conversation, I often think for a long time about what someone actually meant*
32. I usually feel a connection quickly when I'm talking to others
33. I've been pretending to be social for years, but I'm actually not*
34. I automatically know the best thing to say in a conversation
35. People and their behaviour don't interest me much*
36. I've been doing more than I can actually handle all my life*
37. I feel comfortable in a busy environment with a lot of noise, light, and/or smell
38. ~~I can't stand it when others don't appreciate my honesty*~~
39. During a conversation, I am continuously consciously observing how the other person reacts to me*
40. ~~I hardly ever get told that I have to adapt better to others~~
41. ~~I find it easy to share my problems with friends~~
42. I often think out a social event completely in advance*
43. ~~I am fascinated by an idol, for example a pop star or a member of the royal family*~~
44. I become quiet in a conversation with several people*
45. ~~I notice in time when I am thirsty or hungry~~
46. I am satisfied with my relationships with my friends, family, or partner even if they are not perfect
47. ~~In a friendship, I want to have complete control*~~
48. ~~When things go wrong in a relationship, it usually has nothing to do with me~~
49. I usually consciously watch others to see what I should say*
50. I often think out a conversation in advance*
51. ~~If I have too much on my mind, I get angry quickly*~~
52. When I've done too much, I only feel it afterwards*

Original 50 AQ items

With permission of the Autism Research Centre, these items were taken from:

Baron-Cohen, S., Wheelwright, S., Skinner, R., Martin, J., & Clubley, E. (2001). The Autism-Spectrum Quotient (AQ): Evidence from Asperger Syndrome/High-Functioning Autism, Males and Females, Scientists and Mathematicians. *Journal of Autism and Developmental Disorders*, *31*(1), 5–17. https://doi.org/10.1023/A:1005653411471

A formatted version of the original AQ can be freely downloaded from https://www.autismresearchcentre.com/tests/.

* = items are reverse scored; ~~Strikethrough~~ = items were removed after analysis

1. ~~I prefer to do things with others rather than on my own.~~
2. ~~I prefer to do things the same way over and over again.*~~
3. If I try to imagine something, I find it very easy to create a picture in my mind.
4. ~~I frequently get so strongly absorbed in one thing that I lose sight of other things.*~~

5. ~~I often notice small sounds when others do not.*~~
6. ~~I usually notice car number plates or similar strings of information.*~~
7. ~~Other people frequently tell me that what I've said is impolite, even though I think it is polite.*~~
8. When I'm reading a story, I can easily imagine what the characters might look like.
9. I am fascinated by dates.*
10. ~~In a social group, I can easily keep track of several different people's conversations.~~
11. ~~I find social situations easy.~~
12. ~~I tend to notice details that others do not.*~~
13. I would rather go to a library than a party.*
14. ~~I find making up stories easy.~~
15. I find myself drawn more strongly to people than to things.
16. ~~I tend to have very strong interests which I get upset about if I can't pursue.*~~
17. ~~I enjoy social chit-chat.~~
18. ~~When I talk, it isn't always easy for others to get a word in edgeways.*~~
19. I am fascinated by numbers.*
20. When I'm reading a story, I find it difficult to work out the characters' intentions.*
21. ~~I don't particularly enjoy reading fiction.*~~
22. I find it hard to make new friends.*
23. I notice patterns in things all the time.*
24. ~~I would rather go to the theatre than a museum.~~
25. ~~It does not upset me if my daily routine is disturbed.~~
26. I frequently find that I don't know how to keep a conversation going.*
27. I find it easy to "read between the lines" when someone is talking to me.
28. ~~I usually concentrate more on the whole picture, rather than the small details.~~
29. ~~I am not very good at remembering phone numbers.~~
30. ~~I don't usually notice small changes in a situation, or a person's appearance.~~
31. I know how to tell if someone listening to me is getting bored.
32. ~~I find it easy to do more than one thing at once.~~
33. When I talk on the phone, I'm not sure when it's my turn to speak.*
34. I enjoy doing things spontaneously.
35. I am often the last to understand the point of a joke.*
36. I find it easy to work out what someone is thinking or feeling just by looking at their face.
37. ~~If there is an interruption, I can switch back to what I was doing very quickly.~~
38. ~~I am good at social chit-chat.~~
39. ~~People often tell me that I keep going on and on about the same thing.*~~
40. ~~When I was young, I used to enjoy playing games involving pretending with other children.~~
41. I like to collect information about categories of things (e.g. types of car, types of bird, types of train, types of plant, etc.).*

42. I find it difficult to imagine what it would be like to be someone else.*
43. ~~I like to plan any activities I participate in carefully.*~~
44. I enjoy social occasions.
45. I find it difficult to work out people’s intentions.*
46. New situations make me anxious.*
47. I enjoy meeting new people.
48. ~~I am a good diplomat.~~
49. ~~I am not very good at remembering people’s date of birth.~~
50. ~~I find it very easy to play games with children that involve pretending.~~
