# Supplementary material for: Measuring the Autistic Women’s Experience (AWE)
Source: Int J Environ Res Public Health. 2023 Dec 6;20(24):7148. doi: 10.3390/ijerph20247148 (PMC10742621; doi:10.3390/ijerph20247148)
Supplement: Supplementary file 1 [file ijerph-20-07148-s001.zip › Supplementary Materials S2.pdf]

AWE: questionnaire of behaviour and personality

This is a questionnaire about yourself. All information is treated entirely confidentially.

Date of today: / / (day/month/year)

Age: (in years)

Gender: ☐ woman ☐ man ☐ non-binary ☐ transgender woman ☐ transgender man

☐ not listed, state here______________________________________

How do I complete this questionnaire?

Below are a list of statements. Please read each statement very carefully and rate how strongly you agree or disagree with it by marking the box of your chosen answer with an “X”. There are no correct or incorrect answer, or trick questions. Please complete all questions, since otherwise your score is unreliable.

		Definitely agree	Slightly agree	Slightly disagree	Definitely disagree
1.	If I try to imagine something, I find it very easy to create a picture in my mind.				
2.	When I’m reading a story, I can easily imagine what the characters might look like.				
3.	I am fascinated by dates.				
4.	I would rather go to a library than a party.				
5.	I find myself drawn more strongly to people than to things.				
6.	I am fascinated by numbers.				
7.	When I’m reading a story, I find it difficult to work out the characters’ intentions.				
8.	I find it hard to make new friends.				
9.	I notice patterns in things all the time.				

		Definitely agree	Slightly agree	Slightly disagree	Definitely disagree
10.	I frequently find that I don’t know how to keep a conversation going.				
11.	I find it easy to “read between the lines” when someone is talking to me.				
12.	I know how to tell if someone listening to me is getting bored.				
13.	When I talk on the phone, I’m not sure when it’s my turn to speak.				
14.	I enjoy doing things spontaneously.				
15.	I am often the last to understand the point of a joke.				
16.	I find it easy to work out what someone is thinking or feeling just by looking at their face.				
17.	I like to collect information about categories of things (e.g. types of car, types of bird, types of train, types of plant, etc.).				
18.	I find it difficult to imagine what it would be like to be someone else. AQ42				
19.	I enjoy social occasions.				
20.	I find it difficult to work out people’s intentions.				
21.	New situations make me anxious.				
22.	I enjoy meeting new people.				
23.	I go to social events when I don’t want to because it’s expected of me.				

		Definitely agree	Slightly agree	Slightly disagree	Definitely disagree
24.	Contact with other people gives me energy.				
25.	I know everything about one subject, for example about animals, diets, historical periods or upbringing.				
26.	I usually adapt to the other person in a friendship.				
27.	I have enough energy to do normal daily activities.				
28.	I am in touch with my body and rest when I need to.				
29.	I am good at keeping up friendships.				
30.	I feel that I really fit in during a conversation.				
31.	After a few days of doing nothing, I really have to do something				
32.	I notice when I am exhausted or overloaded in time.				
33.	I don’t have to think about how I’m going to act toward others.				
34.	I go to social events when I don’t want to because I’m afraid I won’t belong otherwise.				
35.	I get upset when someone else’s mood isn’t good.				
36.	After a conversation, I often think for a long time about what someone actually meant.				
37.	I usually feel a connection quickly when I’m talking to others.				

		Definitely agree	Slightly agree	Slightly disagree	Definitely disagree
38.	I've been pretending to be social for years, but I'm actually not.				
39.	I automatically know the best thing to say in a conversation.				
40.	People and their behaviour don't interest me much.				
41.	I've been doing more than I can actually handle all my life.				
42.	I feel comfortable in a busy environment with a lot of noise, light, and/or smell.				
43.	During a conversation, I am continuously consciously observing how the other person reacts to me.				
44.	I often think out a social event completely in advance.				
45.	I become quiet in a conversation with several people.				
46.	I am satisfied with my relationships with my friends, family, or partner even if they are not perfect.				
47.	I usually consciously watch others to see what I should say				
48.	I often think out a conversation in advance.				
49.	When I've done too much, I only feel it afterwards.				
