# Supplementary material for: Measuring the Autistic Women’s Experience (AWE)
Source: Int J Environ Res Public Health. 2023 Dec 6;20(24):7148. doi: 10.3390/ijerph20247148 (PMC10742621; doi:10.3390/ijerph20247148)
Supplement: Supplementary file 1 [file ijerph-20-07148-s001.zip › Supplementary Materials S3.pdf]

AWE: score calculation

This is a questionnaire about yourself. All information is treated entirely confidentially.

Date of today: / / (day/month/year)

Age: (in years)

Gender: ☐ woman ☐ man ☐ non-binary ☐ transgender woman ☐ transgender man

☐ not listed, state here____

How do I complete this questionnaire?

Below are a list of statements. Please read each statement very carefully and rate how strongly you agree or disagree with it by marking the box of your chosen answer with an “X”. There are no correct or incorrect answer, or trick questions. Please complete all questions, since otherwise your score is unreliable.

		Definitely agree	Slightly agree	Slightly disagree	Definitely disagree
1.	If I try to imagine something, I find it very easy to create a picture in my mind. AQ3 SI	1	2	3	4
2.	When I’m reading a story, I can easily imagine what the characters might look like. AQ8 SI	1	2	3	4
3.	I am fascinated by dates. AQ9 AD	4	3	2	1
4.	I would rather go to a library than a party. AQ13 ISM	4	3	2	1
5.	I find myself drawn more strongly to people than to things. AQ15 ISM	1	2	3	4
6.	I am fascinated by numbers. AQ19 AD	4	3	2	1
7.	When I’m reading a story, I find it difficult to work out the characters’ intentions. AQ20 SI	4	3	2	1
8.	I find it hard to make new friends. AQ22 SFC	4	3	2	1

		Definitely agree	Slightly agree	Slightly disagree	Definitely disagree
9.	I notice patterns in things all the time. AQ23 AD	4	3	2	1
10.	I frequently find that I don't know how to keep a conversation going. AQ26 SFC	4	3	2	1
11.	I find it easy to "read between the lines" when someone is talking to me. AQ27 SI	1	2	3	4
12.	I know how to tell if someone listening to me is getting bored. AQ31 SI	1	2	3	4
13.	When I talk on the phone, I'm not sure when it's my turn to speak. AQ33 SFC	4	3	2	1
14.	I enjoy doing things spontaneously. AQ34 ISM	1	2	3	4
15.	I am often the last to understand the point of a joke. AQ35 SI	4	3	2	1
16.	I find it easy to work out what someone is thinking or feeling just by looking at their face. AQ36 SI	1	2	3	4
17.	I like to collect information about categories of things (e.g. types of car, types of bird, types of train, types of plant, etc.). AQ41 AD	4	3	2	1
18.	I find it difficult to imagine what it would be like to be someone else. AQ42 SI	4	3	2	1
19.	I enjoy social occasions. AQ44 ISM	1	2	3	4
20.	I find it difficult to work out people's intentions. AQ45 SI	4	3	2	1
21.	New situations make me anxious. AQ46 SFC	4	3	2	1

		Definitely agree	Slightly agree	Slightly disagree	Definitely disagree
22.	I enjoy meeting new people. AQ47 ISM	1	2	3	4
23.	I go to social events when I don't want to because it's expected of me. Novel2 SFC	4	3	2	1
24.	Contact with other people gives me energy. Novel4 ISM	1	2	3	4
25.	I know everything about one subject, for example about animals, diets, historical periods or upbringing. Novel6 AD	4	3	2	1
26.	I usually adapt to the other person in a friendship. Novel8 SFC	4	3	2	1
27.	I have enough energy to do normal daily activities. Novel9 SB	1	2	3	4
28.	I am in touch with my body and rest when I need to. Novel11 SB	1	2	3	4
29.	I am good at keeping up friendships. Novel 17 SFC	1	2	3	4
30.	I feel that I really fit in during a conversation. Novel19 SFC	1	2	3	4
31.	After a few days of doing nothing, I really have to do something. Novel 20 ISM	1	2	3	4
32.	I notice when I am exhausted or overloaded in time. Novel 26 SB	1	2	3	4
33.	I don't have to think about how I'm going to act toward others. Novel27 SFC	1	2	3	4
34.	I go to social events when I don't want to because I'm afraid I won't belong otherwise. Novel28 SFC	4	3	2	1

		Definitely agree	Slightly agree	Slightly disagree	Definitely disagree
35.	I get upset when someone else's mood isn't good. Novel29 SFC	4	3	2	1
36.	After a conversation, I often think for a long time about what someone actually meant. Novel31 SFC	4	3	2	1
37.	I usually feel a connection quickly when I'm talking to others. Novel32 ISM	1	2	3	4
38.	I've been pretending to be social for years, but I'm actually not. Novel33 ISM	4	3	2	1
39.	I automatically know the best thing to say in a conversation. Novel34 SFC	1	2	3	4
40.	People and their behaviour don't interest me much. Novel35 ISM	4	3	2	1
41.	I've been doing more than I can actually handle all my life. Novel36 SB	4	3	2	1
42.	I feel comfortable in a busy environment with a lot of noise, light, and/or smell. Novel37 ISM	1	2	3	4
43.	During a conversation, I am continuously consciously observing how the other person reacts to me. Novel39 SFC	4	3	2	1
44.	I often think out a social event completely in advance. Novel42 SFC	4	3	2	1
45.	I become quiet in a conversation with several people. Novel44 SFC	4	3	2	1
46.	I am satisfied with my relationships with my friends, family, or partner even if they are not perfect. Novel46 SFC	1	2	3	4

		Definitely agree	Slightly agree	Slightly disagree	Definitely disagree
47.	I usually consciously watch others to see what I should say. Novel 49 SFC	4	3	2	1
48.	I often think out a conversation in advance. Novel50 SFC	4	3	2	1
49.	When I've done too much, I only feel it afterwards. Novel52 SB	4	3	2	1

Score calculation of the AWE

The item numbers refer to the final item list of the AWE.

Straight items (“definitely agree” scores 1 point, “slightly agree” scores 2 points, “slightly disagree” scores 3 points, and “definitely disagree” scores 4 points): 1, 2, 5, 11, 12, 14, 16, 19, 22, 24, 27, 28, 29, 30, 31, 32, 33, 37, 39, 42, 46

Reversed items (“definitely agree” scores 4 points, “slightly agree” scores 3 points, “slightly disagree” scores 2 points, and “definitely disagree” scores 1 point): 3, 4, 6, 7, 8, 9, 10, 13, 15, 17, 18, 20, 21, 23, 25, 26, 34, 35, 36, 38, 40, 41, 43, 44, 45, 47, 48, 49

The total AWE score is calculated by summing the score of all the above-mentioned items. The minimum score is 49 and the maximum score is 196, with a score of 123+ predicting an autism diagnosis.

The subscale scores are calculated by summing the scores on all items of a respective scale. The subscales are:

Social functioning and communication (SFC): 8, 10, 13, 21, 23, 26, 29, 30, 33, 34, 35, 36, 39, 43, 44, 45, 46, 47, 48

Initiative and social motivation (ISM): 4, 5, 14, 19, 22, 24, 31, 37, 38, 40, 42

Social intuition (SI): 1, 2, 7, 11, 12, 15, 16, 18, 20

Sensing boundaries (SB): 27, 28, 32, 41, 49

Attention to detail (AD): 3, 6, 9, 17, 25

Dutch norms (EmFASiS 2023)

Women from the general population (N = 237, age 16-82 years)

	VERY LOW	LOW	AVERAGE	HIGH	VERY HIGH
	**Perc≤2.5**	**2.5<Perc≤35**	**35<Perc≤65**	**65<Perc≤97.5**	**Perc>97.5**
**AWE**	≤67	68 - 89	90 - 105	106 - 134	>134
**SFC**	≤24	25 - 37	38 - 44	45 - 62	>62
**ISM**	≤13	14 - 18	19 - 22	23 - 29	>29
**SI**	≤10	11 - 14	15 - 18	19 - 23	>23
**SB**	≤6	7 - 10	11 - 12	13 - 19	>19
**AD**	≤5	6 - 8	9 - 10	11 - 17	>17

Men from the general population (N = 236, age 18-86 years)

	VERY LOW	LOW	AVERAGE	HIGH	VERY HIGH
	**Perc≤2.5**	**2.5<Perc≤35**	**35<Perc≤65**	**65<Perc≤97.5**	**Perc>97.5**
**AWE**	≤67	68 - 92	93 - 105	106 - 132	>132
**SFC**	≤25	26 - 37	38 - 42	43 - 58	>58
**ISM**	≤12	13 - 19	20 - 23	24 - 36	>36
**SI**	≤9	10 - 16	17 - 19	20 - 25	>25
**SB**	≤5	6 - 9	10 - 11	12 - 16	>16
**AD**	≤5	6 - 10	11 - 13	14 - 17	>17

Autistic women (N = 79, age 19-64 years)

	VERY LOW	LOW	AVERAGE	HIGH	VERY HIGH
	**Perc≤2.5**	**2.5<Perc≤35**	**35<Perc≤65**	**65<Perc≤97.5**	**Perc>97.5**
**AWE**	≤95	96 - 136	137 - 147	148 - 171	>171
**SFC**	≤35	36 - 57	58 - 62	63 - 73	>73
**ISM**	≤19	20 - 29	30 - 33	34 - 41	>41
**SI**	≤13	14 - 21	22 - 25	26 – 33	>33
**SB**	≤11	12 - 15	16 - 18	19 - 20	>20
**AD**	≤7	8 - 12	13 - 14	15 - 19	>19

Autistic men (N = 59, age 16-72 years)

	VERY LOW	LOW	AVERAGE	HIGH	VERY HIGH
	**Perc≤2.5**	**2.5<Perc≤35**	**35<Perc≤65**	**65<Perc≤97.5**	**Perc>97.5**
**AWE**	≤90	91 - 125	126 - 143	144 - 171	>171
**SFC**	≤33	34 - 49	50 - 59	60 - 70	>70
**ISM**	≤16	17 - 28	29 - 32	33 - 41	>41
**SI**	≤13	14 - 21	22 - 25	26 – 31	>31
**SB**	≤7	8 - 14	15 - 17	18 - 20	>20
**AD**	≤7	8 - 13	14 - 16	17 - 20	>20
